# Super‐Multiplex Nonlinear Optical Imaging Unscrambles the Statistical Complexity of Cancer Subtypes and Tumor Microenvironment

**DOI:** 10.1002/advs.202104379

**Published:** 2021-12-19

**Authors:** Yanping Li, Binglin Shen, Gengjin Zou, Rui Hu, Ying Pan, Junle Qu, Liwei Liu

**Affiliations:** ^1^ Key Laboratory of Optoelectronic Devices and Systems of Guangdong Province and Ministry of Education College of Physics and Optoelectronic Engineering Shenzhen University Shenzhen 518060 China; ^2^ China–Japan Union Hospital of Jilin University Changchun 130033 China

**Keywords:** cancer research, fluorescence lifetime microscopy, nonlinear optical imaging, tumor microenvironment

## Abstract

Label‐free nonlinear optical imaging (NLOI) has made tremendous inroads toward unscrambling the microcosmic complexity of cancers. However, harmonic and Raman microscopy offers throughput without redox information to reveal metabolic differentiation, and fluorescence lifetime microscopy lacks the vibrational response of molecules to visualize specific molecular constituents such as lipid. Here, a flexible, robust simultaneous multi‐nonlinear imaging and cross‐modality system that combines complementary imaging contrast mechanisms is demonstrated. This system, utilizing multiplexed ultrashort pulses, ingeniously integrates typical nonlinear processes, and high‐dimension lifetime extension in a single setup to enhance the imaging dimensions and quality. Using this system, the authors perform label‐free comprehensive evaluation of clinicopathological tissues of ovarian carcinoma due to its statistical complexity. The results show that the technology provides statistically rich, insightful information with high accuracy, sensitivity, and specificity, in contrast to standard histopathology, and can potentially be a powerful tool for fundamental cancer research and clinical applications.

## Graphical abstract

In this paper, a flexible, robust simultaneous multi‐nonlinear imaging and cross‐modality system that combines complementary imaging contrast mechanisms is demonstrated. This technology provides statistically rich, insightful information with high accuracy, sensitivity, and specificity, in contrast to standard histopathology, and can potentially be a powerful tool for fundamental cancer research and clinical applications.

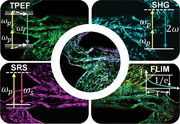

## 1 Introduction

NLOI techniques such as second harmonic generation (SHG),^[^ ^1^, ^2^, ^3^ ^]^ third harmonic generation,^[^ ^4^ ^]^ two‐photon excited fluorescence (TPEF),^[^ ^5^ ^]^ three‐photon excited fluorescence,^[^ ^6^ ^]^ and coherent Raman scattering (CRS)^[^ ^7^, ^8^ ^]^ have emerged as promising tools for investigating complex biochemical processes. The integration of various NLOI techniques allows accurate multidimensional photophysical information to be obtained and rapid clinicopathological examination to be performed and has proven to be a pioneering trend^[^ ^9^, ^10^, ^11^, ^12^, ^13^, ^14^, ^15^, ^16^, ^17^, ^18^, ^19^, ^20^ ^]^ despite some significant challenges. The primary challenge arises from the requirement of different excitation conditions for fluorescence and harmonic processes versus CRS. Excitation with a femtosecond laser for the TPEF and SHG modalities results in high photon yield in tissue and cell samples but to poor spectral resolution for CRS. For instance, the spectral bandwidth of a commercial Ti:sapphire femtosecond laser is typically 8–15 nm. Such a bandwidth can resolve the molecule vibration at a resolution of only ≈250 cm^−1^. However, directly implementing a narrow linewidth picosecond laser and an optical parametric oscillator as a laser source of CRS may inevitably cause low excitation efficiency for TPEF and SHG, especially for two‐photon excitation fluorescence lifetime imaging microscopy (TP‐FLIM) based on single photon counting.^[^ ^11^ ^]^ Another challenge is the requirements of obtaining photons per pixel (efficient optics and electronics)^[^ ^21^ ^]^ sufficient for determining the lifetime decay and achieving a high image quality and of spectral scanning to obtain a whole Raman spectrum. These lengthy acquisitions are worsened by the relatively low photon conversion efficiency of high‐order nonlinear processes and the presence of background interference including ambient light, readout noise, dark current, and shot noise. To date, the simultaneous integration of TPEF, SHG, FLIM, and CRS imaging modalities has not yet been realized, probably due to excitation conflicts and efficiency insufficiency. Current multiplexed imaging techniques rely primarily on iridescent fluorescent nanoprobes and dyes to discern diverse constituents and have shown great accomplishments in the biosciences.^[^ ^22^, ^23^, ^24^, ^25^, ^26^, ^27^, ^28^, ^29^ ^]^ However, the biomedical optics community has been challenged with the requirements of accurate, comprehensive, and quantitative information extraction beyond high resolution, deeper penetration, and fast imaging from biologists, pathologists, and physicians. There are increasing demands for the development of methods that combine the imaging modalities of these nonlinear processes to uncover the mysteries of embryonic growth, brain function, cancer infiltration, etc.

To overcome the above challenges, we developed a simultaneous multi‐nonlinear imaging and lifetime evaluation (SMILE) platform that integrates fast Fourier transform (FFT)‐SHG, spectral‐focusing stimulated Raman scattering (SRS), TPEF, and phasor‐empowered TP‐FLIM on a home‐built two‐photon excitation microscope. Our technique can acquire complementary and multidimensional information with high contrast and precision from unstained tissue samples using a large‐area photodiode (PD, 1‐cm^2^ active area), a photomultiplier tube (PMT), and a high‐speed PMT (picosecond rise time). The Raman spectrum of the sample is collected by regulating the instantaneous frequency difference between the pump beam and the Stokes beam. Meanwhile, this time window is exploited for photon accumulation to construct the TP fluorescence lifetime decay based on time‐correlated single photon counting (TCSPC) and to attain SHG with a high signal‐to‐noise ratio (SNR). Therefore, this approach has advantages in imaging speed and quality.

The SMILE imaging technique presented in this work has been proven to be able to retrieve multidimensional optical characteristics for high‐precision diagnosis and complementary medical research on human ovarian carcinomas. Specifically, SHG signals that occur in noncentrosymmetric materials and hyperpolarizable biological molecules are specific to collagen, microtubules, and myosin. TPEF allows visualization of various endogenous fluorophores (NADH, FAD)^[^ ^5^ ^]^ to analyze metabolic activity, and their fluorescence lifetime sheds light on metabolic changes associated with carcinogenesis.^[^ ^30^ ^]^ CRS, especially unbiased SRS,^[^ ^8^, ^31^, ^32^ ^]^ is capable of biochemical visualization and quantification. The SMILE system, relying on various contrast mechanisms, can be used to precisely and comprehensively identify and study the molecular and structural specificity of tumor tissue specimens. The synergistic effect of the multiple nonlinear modalities and their corresponding biometric analysis methods demonstrate the powerful capability of this technique for classification of different cancer types and analysis of tumor microenvironment (TME) characteristics and metabolism at the cellular scale.

## 2 Results

### 2.1 Principle of the SMILE System

**Figure 1:**
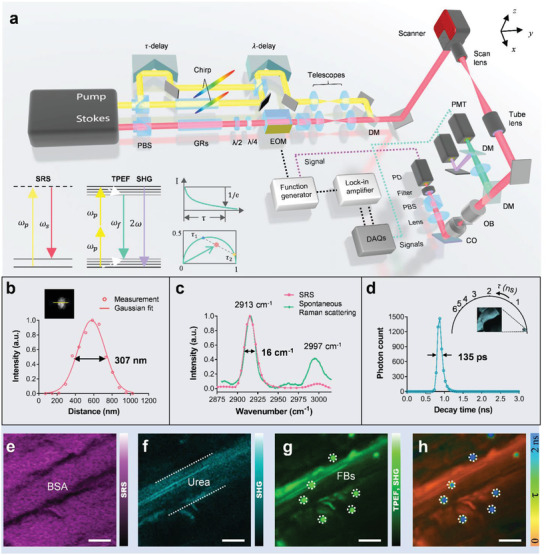
Schematic and performance of the SMILE system. a) Schematic of the SMILE optical system. See complete details in Experimental Section. Bottom left shows Jablonski diagrams of SRS, SHG, and TPEF with its lifetime decay and phasor transformation. b) Optical resolution of the system measured using small diameter fluorescent beads. The cross‐sectional profile in the lateral dimensions is shown with Gaussian fits. c) Spectral resolution of SRS measured as the full width at half maximum (FWHM) of the narrowest Raman peak in the dimethyl sulfoxide (DMSO) spectrum. d) Temporal resolution of TP‐FLIM measured using urea powders. The phasor plot gives the lifetime limit. (e)–(h) correspond to simultaneous contrast imaging by SRS, SHG, TPEF, and TP‐FLIM. CO, condenser; DAQ, data acquisition system; DM, dichroic mirror; EOM, electro–optic modulator; GR, glass rod; *λ*/2, half‐wave plate; OB, objective; PBS, polarizing beam splitter; PD photodiode; PMT, photomultiplier tube; *λ*/4, quarter‐wave plate. Scale bars = 10 µm.

The SMILE system offers a powerful multimodal optical approach to simultaneously explore diverse optical characteristics and molecular mechanisms of specimens. The system mainly consists of a broadly tunable dual‐output femtosecond laser, a complex multithreading optical path arrangement, a homemade two‐photon excitation microscope, and a user‐friendly synchronous control interface, as shown in **Figure** **1**a. On one hand, regarding the optical path of excitation, spectral‐focusing SRS is excited with two synchronized picosecond pulses (pump, *ω*_p_; Stokes, *ω*_s_) chirped from femtosecond pulses by glass rods. The high‐accuracy motorized linear stage (*λ*‐delay) in the pump path regulates the time delay to effectuate temporal overlap and spectral scanning. The intensity of the Stokes beam is modulated by a resonant electro–optic modulator (EOM). On the other hand, due to the decreased nonlinear excitation efficiency when using a picosecond pulse for excitation of TPEF (*ω*_*f*_), SHG (2*ω*), and FLIM (*τ*) imaging, we route a vertically polarized femtosecond pulse by a polarizing beam splitter (PBS) before chirping (Figure 1a). To avoid excitation interference (see the discussion below), another motorized linear stage (*τ*‐delay) is inserted in the femtosecond beam to adjust its time interval with the picosecond pulses. All the pulses are exquisitely temporally arranged, spatially collinearly, and then sent to the two‐photon excitation microscope. Complete details of the system can be found in the Experimental Section, and synchronization of logical relationships among the local instruments, including the detectors, data acquisitions (DAQs), laser, and scanner, for simultaneous multimodal imaging in the SMILE platform is detailed in the Experimental Section and Figure S1, Supporting Information.

Notably, the combination of the femtosecond pulse for efficient SHG and TP‐FLIM and picosecond pulses for hyperspectral SRS exhibits excitation interference and emission crosstalk, which can result in low efficiency and inaccuracy in data acquisition. The emission crosstalk can be easily solved using contrary detection mechanisms (transmission for SRS and reflection for TPEF and SHG) and suitable filter combinations. The excitation interference, originating from simultaneous implementation of the three ultrashort pulses, raises some significant challenges. The first challenge, termed the spatial issue, is to achieve complete spatial overlap of the three beams. Misalignment (Figure S2c, Supporting Information) of the pump and Stokes beams can cause nonuniformity in the SRS image (Figure S2a, Supporting Information) compared to the reference image (Figure S2b, Supporting Information). Misalignment of the femtosecond and picosecond beams can result in ghosting in the TPEF (FLIM) image (Figure S2d, Supporting Information). This misalignment will lead to drops in excitation efficiency for SRS and spatial resolution for TPEF. The second challenge is the demodulation issue. The femtosecond pulse for TPEF (FLIM) with the same wavelength as the pump beam can result in a significant degradation in stimultaed Raman loss (SRL) demodulation (Figure S2f, Supporting Information) even though it is temporally isolated from the Stokes pulse (i.e., it does not interact with the molecule vibrations). To avoid this, we inset a PBS into the transmission detection path to separate the femtosecond pulse by its orthogonal polarization (Figure S2e, Supporting Information) before the beam enters the PD. Then, the resulting image (Figure S2b, Supporting Information) has no obvious decline compared to the pristine reference image without the femtosecond beam. The third challenge is the temporal issue. For SRS, the pump and Stokes pulses should be temporally overlapped for spectral focusing, which can be realized by adjusting the *λ*‐delay. However, for TP‐FLIM, when combined with SRS, there is a tradeoff between photobleaching (as well as the thermal effect) and excitation interference when simultaneously implementing the femtosecond pulse and picosecond pulses. Temporal overlap of these pulses can lead to high heat storage and extremely low spectrum resolution, whereas staggering of them can result in convolution of the fluorescence lifetime. This convolution can be attributed to the mixture of fluorescence decays induced by the femtosecond pulse and picosecond pulses (although their stimulating efficiencies are different^[^ ^11^ ^]^). The two decays, when mismatched in time, can result in a considerable inaccuracy in fluorescence lifetime determination (Figure S2g, Supporting Information). Therefore, under comprehensive consideration, we adjust the *τ*‐delay to realize an ≈50–100 ps temporal interval between the femtosecond and picosecond pulses. This interval is selected to be much larger than the pulse width of the picosecond pulses to reduce thermal storage and photobleaching and less than the temporal resolution for TP‐FLIM to avoid lifetime convolution (Figure S2h, Supporting Information).

Calibrations and performances of the system are given in Experimental Section, Figure S3, Supporting Information, and Figure 1b–d. Regarding the emission spectrum of FAD, the spatial resolution measured as the FWHM of the cross section (Figure 1b) of fluorescent beads (FBs) is ≈307 nm for 550‐nm collection and 1.20 NA objective, sufficiently high for revealing collagen arrangements. We capture the Raman spectrum of DMSO at a wavenumber resolution of 3.5 cm^−1^ to obtain the spectral resolution of SRS. This spectral resolution, which is quantified to be ≈16 cm^−1^ (Figure 1c), is sufficient to separate the Raman peaks of lipids and proteins based on their intrinsic molecular vibrations. We also use mixed excitation pulses to image urea crystals with strong SHG signals. Notably, the fluorescence lifetime shows very little dependence on the wavelength of the exciting radiation. The temporal resolution obtained from the decay curve of the SHG signal of the urea reaches 135 ps (Figure 1d), matching the instrument response function (IRF, 130 ps) of the high‐speed PMT. Nonetheless, the fit‐free phasor plot method^[^ ^33^ ^]^ provides a higher accuracy value of 70 ps that agrees more with the instantaneous feature. This high resolution enables TP‐FLIM enhanced by the phasor approach to accurately feature the metabolic processes of oxidative phosphorylation and glycolysis in cellular aerobic and anaerobic respiration. We use the system to image a multiple component specimen containing bovine serum albumin (BSA), urea, and FBs (Figure 1e–h and Figure S4, Supporting Information). Consequently, different modalities can visualize different substances. For example, SRS reveals BSA (Figure 1e, collected by 825/150 nm), SHG reveals urea (Figure 1f, collected by 400/10 nm), and TP‐FLIM distinguishes urea and FBs (Figure 1h, collected by 550/80 nm) via their discrepant fluorescence lifetime despite the approximate fluorescence intensities (Figure 1g). This demonstrates the contrast and specificity features of the diverse modalities.

### 2.2 Multimodal Imaging of Clinicopathological Tissues

**Figure 2:**
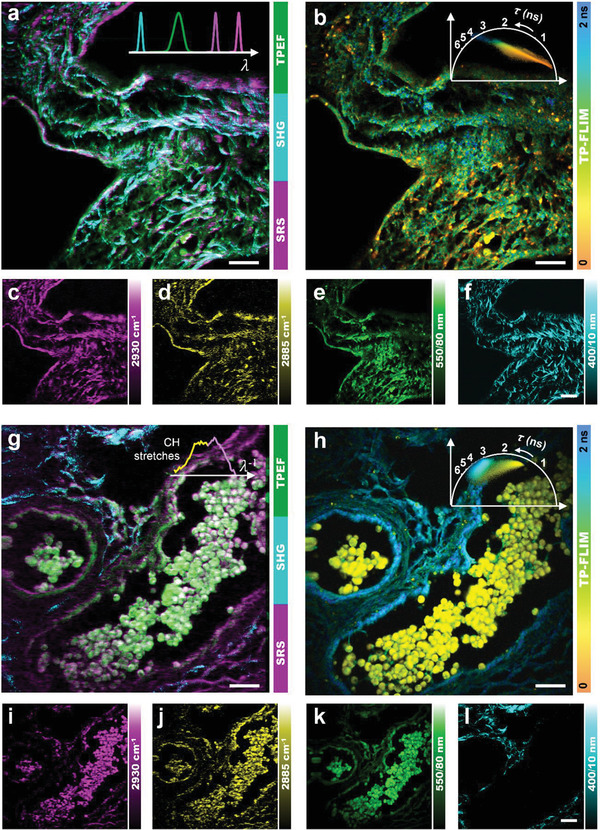
Multimodal imaging of clinicopathological ovarian tissues. Also see Figure S6, Supporting Information. a,g) Pseudocolor presentation merging cyan (SHG of collagen), green (TPEF of FAD), and magenta (SRS of protein). b,h) TP‐FLIM image of FAD collected at 550 nm with phasor plots given in the insets. c,i) SRS image of proteins at 2930 cm^−1^. d,j) SRS image of lipids at 2885 cm^−1^. e,k) TPEF image of FAD collected at 550 nm. f,l) SHG image of collagen fibers collected at 400 nm. (a)–(f) Correspond to normal ovarian tissue and (g)–(l) correspond to SBT. The profiles in (a) indicate the wavelength diversity of SHG, TPEF, pump, and Stokes pulses. The profile in (g) depicts the Raman spectrum. The color bar in (a) and (g) represent the pseudocolor of the three modalities, the color bar in (b) and (h) represent the lifetime range, and those in the other images represent the normalized intensity. Scale bars = 20 µm.

We demonstrate the application of the SMILE system by simultaneous multimodal imaging of intraoperative frozen sections of ovarian cancer. As the most fatal gynecological malignancy, ovarian cancer is the fifth leading cause of cancer‐related death in women. More than 70% of patients are diagnosed with stage III or IV (International Federation of Gynecology and Obstetrics, FIGO) due to its generally vague symptoms and the lack of effective screening methods for the diagnosis of early ovarian cancer.^[^ ^34^ ^]^ We first perform a standard pathological examination, that is, hematoxylin and eosin (H&E) histopathology, to differentiate different types of tumors (Note S1 and Figure S5, Supporting Information). These ovarian cancer subtypes include serous borderline tumor (SBT), endometrioid carcinoma (EC), high‐grade serous carcinoma (HGSC), and mucinous carcinoma (MC). The restricted preparation carried out by highly trained technicians provides preliminary diagnosis interpretation by an experienced pathologist. Then, we acquire four‐modality optical images of the unstained ovarian tissue specimens (**Figure** **2**) using the SMILE platform. Note that we used a narrower band filter (550/40 nm, Table S1, Supporting Information) compared to Figure 1b,h to collect TPEF while avoid SHG mixing to focus on the analysis of free and bound FAD. Consequently, diverse modalities reveal discrepant tissue components and provide much valuable information in the TME. Each imaging modality can provide its featured information, such as energy metabolism associated with the redox reaction (Figure 2b,h) based on the diversities of component lifetime and ratio of bound FAD, molecule‐scale lipid metabolism mapping (Figure 2d,j) based on the Raman peak shifts of lipid, and tumor‐invasion‐associated collagen signals (Figure 2f,l). These features, as well as the latent higher‐level information, can be extracted using the introduced algorithms and are analyzed in detail below.

### 2.3 Quantification of Tumor‐Associated Collagen Signature

SHG imaging revealing the relative changes in the extracellular matrix (ECM) has already been proven to have potential applicability for cancer diagnosis.^[^ ^1^, ^2^ ^]^ As type I collagen fibers are the major structural component of the ovarian stroma, SHG is an appropriate method to characterize the morphological and structural characteristics of the ECM. We compare a normal human ovarian specimen and four subtypes of ovarian tumor in the collagen‐rich areas. The merged image of the TPEF and SHG direct views represents the epithelium/stromal interface (see Note S2 and Figure S7, Supporting Information), and the individual SHG view reveals the matrix organization, density, and collagen fiber orientations in the ovarian stroma. Two evaluation methods are used to quantify the specificity of the ECM: tumor‐associated collagen signature (TACS) and aspect ratio (AR) analyses.^[^ ^35^ ^]^ TACSs are classified into three types:^[^ ^36^, ^37^ ^]^ TACS‐1 to TACS‐3 (Note S2, Supporting Information). AR analysis quantifies the anisotropy of the ECM using the AR of the bidimensional intensity distribution in the FFT images.

**Figure 3:**
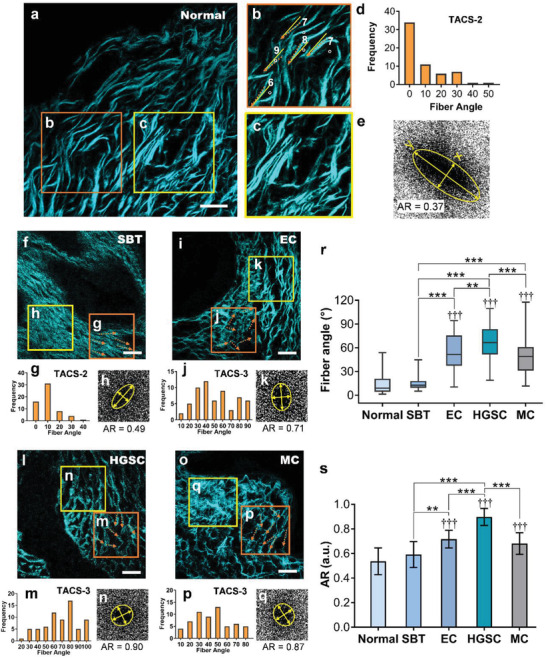
Collagen quantitative analysis in the SHG modality. SHG images of a) normal ovarian, f) SBT, i) EC, l) HGSC, and o) MC tissues. b) Orientation of collagen fiber proliferation (yellow arrows) with regard to the epithelium alignment (orange dashed lines), which quantifies the TACS. c) region of interest (ROI) in (a) for Fourier transformation. Angle frequency of TACSs (ROIs in the orange border) for d) normal ovarian, g) SBT, j) EC, m) HGSC, and p) MC tissues. FFT of ROIs in the yellow border for e) normal ovarian, h) SBT, k) EC, n) HGSC, and q) MC tissues to obtain the AR values by ellipse fitting (yellow circle). *x*: minor axis; *y*: major axis. r) Statistical analysis of collagen orientations in different cancer types based on the principal distribution of the collagen fiber angle (*n* = 60). s) Statistical analysis of the AR in different cancer types (*n* = 10). An unpaired two‐tailed *t*‐test is applied, and the mean ± standard deviation (SD) is shown in (r) and (s). † indicates comparisons with the normal tissue. * indicates comparisons between line‐connected columns. †, *: *p* < 0.05; ††, **: *p* < 0.01; †††, ***: *p* < 0.001. Scale bars = 20 µm.

The SHG characteristics in **Figure** **3** demonstrate that different types of cancer tissues have distinctive collagen properties. For example, normal ovarian collagen features a loose, supple, and regular shape (Figure 3a); SBT tissue contains highly fibrotic and thick collagen fibers that stack neatly (Figure 3f); the HGSC morphology exhibits random arrangement of fibers in multiple directions (Figure 3l); and EC and MC collagen consists of a sparser, shorter, rhombic reticular fiber structure (Figure 3i,o). The collagen fiber angles relative to the epithelium for different types of cancer tissues are detailed in Note S2, Supporting Information, and statistically quantified in Figure 3r. The comparison shows significant differences between normal tissues and cancer tissues. The growth and extension of collagen fibers in ovarian cancers except SBT are statistically at a larger angle relative to the epithelial stroma boundary than those for normal tissue (*p* < 0.0001 for EC, HGSC, and MC with the normal tissue and *p* = 0.518 for SBT with the normal tissue). SBT exhibits an extremely small *p* value of less than 0.0001 in fiber angle compared with EC, HGSC, or MC (Figure 3r). HGSC can obviously be discriminated from MC (*p* < 0.0001) but exhibits a lower characteristic diversity with EC (*p* = 0.0055). The AR value of the normal ovarian tissue is also significantly different from that of most ovarian carcinomas (*p* < 0.01 for EC, HGSC, and MC) but presents a similar value to SBT (*p* = 0.2794) containing a set of aligned fibers (Figure 3s). See more discussions in Note S2, Supporting Information. The same conclusion applies to other test images (Figure S8, Supporting Information). These results confirm the power of SHG imaging and quantification in classifying different subtypes of ovarian cancer and studying its statistical complexity. Nevertheless, this modality still exhibits a low ability to discriminate normal and SBT classes and cannot be applied in collagen‐deficient areas. This greatly limits its scope of application.

### 2.4 Quantification of Protein and Lipid Metabolism within the Tumor Microenvironment

**Figure 4:**
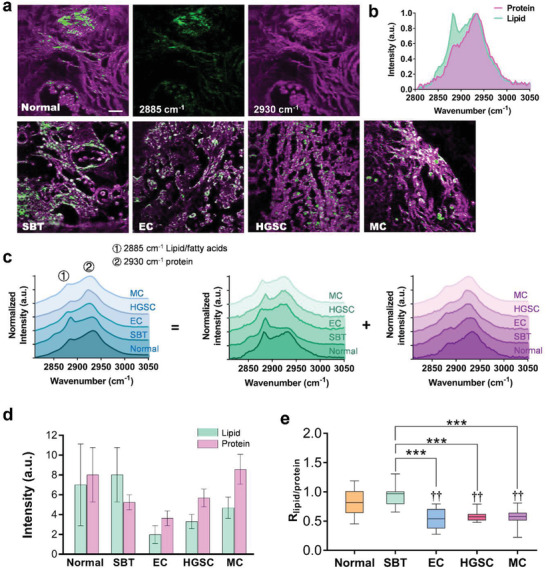
Hyperspectral SRS imaging of lipids and proteins in cancerous ovarian tissue. Also see Figure S9, Supporting Information. a) Overlay image of lipids (green) and proteins (magenta) in different pathological types of ovarian tissue. b) Line plot corresponding to the hyperspectral stack of 80 images ranging from 2810 to 3050 cm^−1^. c) Overall SRS spectra and spectral decomposition of lipids (green) and proteins (magenta) for the entire FOV. d) Statistical intensities of lipids and proteins and e) their intensity ratio, *R*_lipid/protein_, for the pathological tissues (*n* = 10). An unpaired two‐tailed *t*‐test is applied in (e), and the mean ± SD is shown in (d) and (e). ††: *p* < 0.01; ***: *p* < 0.001. Scale bars = 20 µm.

As a contrasting and a supplemental technique to SHG, SRS provides an efficient way to detect molecular vibrations and translate the biochemical features from the signal strength, peak position, and relative peak shift of the Raman spectra.^[^ ^38^, ^39^, ^40^ ^]^ We perform unbiased, hyperspectral SRS imaging on the collagen‐deficient, lipid‐rich regions of the pathological tissues to demonstrate the protein and lipid metabolism features of our system. The SRS unit acquires the Raman spectra at a resolution of 3.5 cm^−1^ in the high‐frequency CH stretch region, and the resulting sequential images (**Figure** **4**a) are improved using our previous self‐reinforcing global spectral denoising (GSD, see Experimental Section) algorithm.^[^ ^41^ ^]^ Then, we use the multivariate curve resolution (MCR) algorithm^[^ ^42^ ^]^ to reconstruct the spatial distribution maps of lipids and proteins and extract the optimized decomposed spectra (Figure 4b). The proteins in the tissues primarily correspond to a symmetric CH_3_ stretching mode at 2930 cm^−1^, while the lipids and fatty acids exhibit a strong peak at 2885 cm^−1^.^[^ ^32^ ^]^

Figure 4c reveals the differences in Raman peaks of unsaturated and saturated fat between the normal ovary and different ovarian cancer types. For instance, the Raman peak of saturated fat at 2850 cm^−1^ in normal and SBT tissues is more obvious than that in other malignant tumor tissues, and the peak at 2885 cm^−1^ in normal, SBT, and HGSC tissues shifts to ≈2878 cm^−1^ (unsaturated fat) in EC and MC tissues. This suggests the potential for the Raman peaks of lipids at 2850 and 2885 cm^−1^ to be efficient optical markers for ovarian cancer diagnosis.^[^ ^40^ ^]^ Moreover, Figure 4d reveals that the Raman signal intensities of protein and lipid contents vary greatly in normal tissue and cancer tissues (see the analysis in Note S3, Supporting Information), demonstrating their different metabolic performances. However, this conclusion based on absolute strength could be susceptible to the sample preparation processes and subjectively selected imaging areas. Thus, we further extract the difference using the content ratio of lipids and proteins. As shown in Figure 4e, the normal, SBT, and malignant tumor tissues exhibit statistically significant differences in protein and lipid fractions (normal: 0.83 ± 0.07; SBT: 0.94 ± 0.06; EC: 0.53 ± 0.05; HGSC: 0.58 ± 0.03; MC: 0.56 ± 0.05, mean ± SD), which can be attributed to the dysregulation of lipolysis and ECM reorganization (involving serum albumin) during cancer invasion.^[^ ^11^, ^43^ ^]^ These lipid/protein metabolism changes indicate the pathological transformation of early tumors to highly life‐threatening malignancies. In brief, SRS imaging allows superior molecular‐scale analysis of normal, SBT, and malignant tumor tissues and hence fills in the gap in SHG imaging, which exhibits a low ability to resolve normal and SBT tissues. Nonetheless, the SRS modality also has deficiencies in distinguishing ovarian carcinomas, especially EC, HGSC, and MC, because they have hardly any differences in spectral shift and lipid and protein content ratio and have a low analytical ability in lipid‐deficient regions.

### 2.5 Quantification of the Lifetime and Contribution of Protein‐Bound and Free FAD

**Figure 5:**
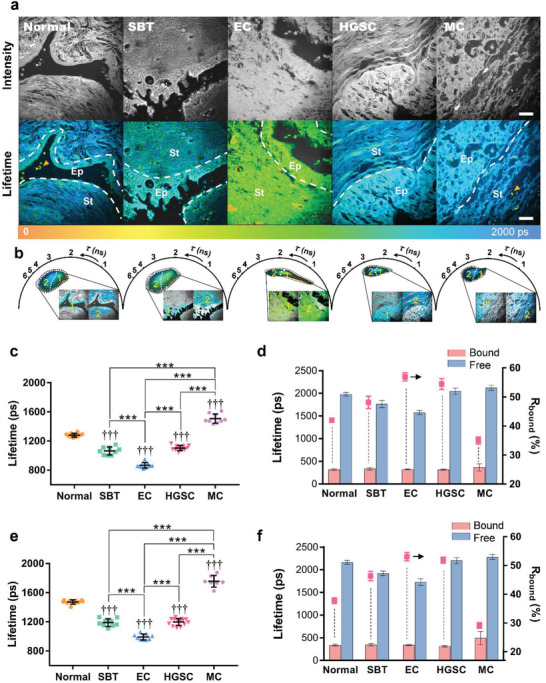
FLIM and phasor analysis of energy metabolism in ovarian tissue. Also see Figure S10, Supporting Information. a) Fluorescence intensity (grayscale) and lifetime (pseudocolor) images of endogenous fluorescence in different pathological types of ovarian tissue. Ep: epithelium; St: stroma. b) Phasor‐mapped images of the epithelium and stroma based on the FLIM data in (a). The cluster areas (1 and 2) correspond to Ep and St in the inset pseudocolor images. Statistical mean fluorescence lifetime (*τ*_m_) in the c) Ep and e) St. Statistical component lifetime (*τ*_1_ and *τ*_2_) and ratio (*R*_bound_) in the d) Ep and f) St, where pink (*τ*_1_) and blue (*τ*_2_) histograms represent the bicomponent decomposition of bound and free FAD, respectively and the magenta dots indicates *R*_bound_. Sample sizes for each statistical analyses are specified in Table S3, Supporting Information. An unpaired two‐tailed *t*‐test is applied in (c) and (e), and the mean ± SD is shown in (c) and (d). †††, ***: *p* < 0.001. Scale bars = 20 µm.

Tumor formation is a multistep complicated process involving gene alterations in epithelial cells, and epithelial–stromal interactions affect the tumorigenesis and migration of tumor cells,^[^ ^35^, ^44^ ^]^ which can be characterized by the fluorescence lifetime of endogenous fluorophores.^[^ ^45^, ^46^ ^]^ To complement the imaging capability of the system in areas with less collagen and lipids, as well as analyze the metabolic specificity for tumor pathological diagnosis and identification,^[^ ^47^, ^48^ ^]^ we perform TP‐FLIM imaging on unstained ovarian tissues (**Figure** **5**a). To distinguish different lifetime components in these tissues rapidly, we introduce fit‐free phasor approach that can provide a 2D distribution of lifetime populations using our previous bicomponent cluster method^[^ ^11^ ^]^ (see Experimental Section and Figure S11, Supporting Information).

The phasor views of TP‐FLIM in Figure 5b show that the epithelium and stroma of the ovary are distinguished via the lifetime heterogeneity in the phasor plots. The fluorescence lifetime of FAD in stromal cells has a higher weighted mean fluorescence lifetime (*τ*_m_) compared to epithelial cancer cells, which indicates the invasion pathway in epithelial ovarian cancers from epithelial tumor cells into the ECM. The analysis in Note S4, Supporting Information, reveals that different ovarian cancer types exhibit extremely significant differences in *τ*_m_ (*p* < 0.001) of the epithelium (Figure 5c) and stroma (Figure 5e). Since *τ*_m_ is contributed by bicomponent: bound FAD with short lifetime and free FAD with long lifetime, we further calculated the component lifetime and ratio of free and bound FAD in the epithelium (Figure 5d) and stroma (Figure 5f) of the ovarian tissues using biexponential analysis.^[^ ^11^, ^33^ ^]^ Compared to normal ovarian tissues, a significant decrease in the lifetime of free FAD is observed in the epithelium and stroma of SBT and EC tissues, yet opposite variations are observed in HGSC and MC tissues because of the diverse glycolysis characteristics and redox processes in different cancer tissues. Associated largely with the oxidative phosphorylation processes,^[^ ^49^ ^]^ fractions of bound FAD (*R*_bound_) localized in mitochondria exhibit great differences between normal, SBT, EC, HGSC, and MC tissues. For instance, *R*_bound_ for the stroma increases from 37.7% in the normal tissue to 53% in EC tissue and then reversely decreases back to 29.1% in MC tissue (Table S3, Supporting Information), indicating the high metabolic diversities among these ovarian carcinomas. These metabolic features can be attributed to the redox changes of oxidative phosphorylation and glycolysis in the TME during cancerization and be another endogenous optical biomarker for cancer characterization. Consequently, unlike SHG being specific to TAC and SRS focusing on the molecular vibration of fat, TP‐FLIM can accurately reveal the metabolism changes by the lifetime and component discrepancy of the endogenous metabolic substances.

## 3 Discussion

Label‐free NLOI plays an essential role in understanding the cause, development, and metastasis of cancer and provides efficient diagnostic strategies for indiscernible cancers.^[^ ^11^, ^50^, ^51^ ^]^ In this work, we present a label‐free multimodal NLOI system that captures the autofluorescence intensity and lifetime, harmonic generation, and Raman spectral signals. In contrast to previous multimodal microscopes, the advantage of the SMILE system is that by using multiplexed ultrashort pulses for efficient excitation, multidimensional, impalpable feature information can be simultaneously collected to reveal the statistical complexity of intractable cancers. This platform, featuring high sensitivity and specificity, noninvasiveness, and versatility, is an attractive alternative or complementary approach for assisting in rapid disease diagnosis and superseding surgical frozen section histopathological analysis.

Additionally, the optical signatures of each module in the SMILE microscope can be quantified using the corresponding analysis methods to objectively evaluate structural, molecular, and metabolic properties in cancers. Specifically, the morphological descriptions merging the SHG and TPEF modalities effectively distinguish epithelial and stromal structures in tissues. The SHG imaging enhanced by TACS and FFT analysis further reveals the arrangement of collagen in the stroma, which provides an indicator associated with cancer infiltration. The lifetime of free and bound metabolites detected by TP‐FLIM combined with the phasor approach indicates the metabolic shift from oxidative phosphorylation to glycolysis and, possibly, to cancerous behavior for different stages or types of cancers. The Raman spectrum measured by the SRS modality offers an approach to monitor macromolecular metabolism during cancerization, which is often accompanied by protein and lipid component deviation. These imaging modalities are contrasting and complementary due to the difference in their major target substances. Moreover, extension of CRS from lipid region to fingerprint regions can be realized by tuning the pump wavelength to 880–950 nm; extension of FLIM to interrogate NADH can be achieved by implementing a collect filter around 450 nm;^[^ ^11^ ^]^ extension of samples to live cells/tissues can be mitigated by the introduced self‐reinforcing algorithm.^[^ ^41^ ^]^ Therefore, the SMILE system enables direct visualization and accurate quantification of multidimensional information with higher speed, contrast, and SNR compared to modality‐isolated imaging and sequential multimodal imaging.

Nonetheless, there are still some technical barriers to clinical diagnosis applications. For example, although SRS imaging is not influenced by a nonresonant background compared to coherent anti‐Stokes Raman scattering,^[^ ^7^ ^]^ the commonly used transmission detection method impedes its applications in in vivo imaging (except for thin tissues such as ears). In contrast, the implementation of epi‐detection to partially collect the scattered excitation light can result in a low efficiency in SRL demodulation and hence greatly limits the imaging speed. Moreover, examination of tissue section at a small scale may be complicated by intratumor heterogeneity, which can be alleviated by collecting and calculating more statistics. Nonetheless, the requirement for elaborative analysis of large amounts of statistical data affects the readability of the conclusions and generalization of the system. This may be solved by the introduction of AI algorithms, which have made tremendous inroads in image quality enhancement, data processing, and result analysis and readout.^[^ ^52^ ^]^ Thus, AI analysis can be applied in conjunction with our platform for further foreseeable improvements. Finally, highly expensive and complicated system may be unaffordable and inconvenient in a clinical setting. We expect simpler, more compact, and more flexible systems, such as endoscopic,^[^ ^53^ ^]^ fiber‐based, and portable setups, which can be cost‐reducing and easier to use for pathologists and physicians. Therefore, a convenient and simplified design and analysis method can be a future direction of the technique.

To conclude, the complementary and synergistic effects of the imaging modalities demonstrate the sufficiently high capability of the system to achieve direct subcellular visualization and molecule‐scale quantitative analysis. The simultaneous imaging accelerates the process of real‐time intraoperative evaluation and enables comprehensive evaluation of pathological features of cancers compared to conventional histological assessments. With its superior spatial, spectral, and temporal resolution to uncover the cell structure, behavior, and function, SMILE, which provides enormous amounts of complex information, can potentially be a powerful tool to assist in rapid clinical diagnosis and biomedical research.

## 4 Experimental Section

### Sample Preparation

All tissue samples were collected from 30 patients in China–Japan Union Hospital of Jilin University, with approval by the Ethics Committee and informed written consent. All patients with suspected and confirmed ovarian cancers were approached for recruitment. Tissue samples were surgically removed, snap frozen in liquid nitrogen and stored at −80 °C until being cut into 5 µm sections for unstained and stained (histology) sectional applications using a freezing microtome (CM1850, Leica, Germany). Histological sections with H&E staining were obtained for experienced gynecological oncologists to conduct histological identification and classification. Unstained frozen tissue sections were simply covered with a coverslip, imaged by the system, and preserved in a −80 °C refrigerator.

### Optical Setups

For the spectral‐focusing SRS experiment setup, a broadly tunable dual‐output femtosecond laser (pulse width: 100 fs, repetition rate: 80 MHz, Chameleon Discovery, Coherent) provides two synchronized output beams: a pump beam at 800 nm with a pulse width of 126 fs and a Stokes beam at 1040 nm with a pulse width of 107 fs measured using an autocorrelator (pulseCheck, APE). The pump and Stokes beams were chirped to 2.0 and 1.8 ps, respectively, using SF75 glass rods. The total group delay dispersions (GDDs) induced in the spectral phases were calculated to be 80488 fs^2^ for the pump beam and 72742 fs^2^ for the Stokes beam,^[^ ^54^ ^]^ while an additional GDD of −7000 fs^2^ was compensated for in the pump beam within the laser to achieve the same linear chirp parameter *β* of the two beams. The spectral resolution of SRS was measured to be 16 cm^−1^, which was sufficient to separate the Raman peaks of lipids and proteins in the CH regions. A motorized linear stage (accuracy: ±1.7 µm; M‐ILS250CC, Newport) was inserted into the pump beam for temporal overlap of two pulses and spectral scanning. The intensity of the Stokes beam was modulated by a resonant EOM (EO‐AM‐R‐20‐C2, Thorlabs) at 20 MHz. To enhance the modulation depth with a relatively low‐level driving voltage, a half‐wave plate and a quarter‐wave plate were used to modify the Stokes beam by circular polarization. A driving voltage range of only −1/2 to 1/2 V_*π*_ was sufficient to fully modulate the amplitude of the Stokes beam in this polarization state. The pump and Stokes beams were then collinearly aligned through a DM (SP950, Thorlabs) and sent to a home‐built laser scanning microscope. A 60× water immersion objective lens (UPLSAPO, 60×, 1.2 NA, Olympus) focused the beams on the sample, and an oil condenser (U‐ACC, 1.4 NA, Olympus) collected the transmitted light. The modulated Stokes beam was filtered out through a hard‐coated bandpass filter (825/150 nm, Chroma) with optical density > 6, and the pump beam was detected by a PD (FDS1010, Thorlabs) driven by a home‐built transimpedance amplifier with a bandwidth of 100 MHz. The SRS signal was eventually demodulated as the SRL using a lock‐in amplifier (HF2PLL, Zurich Instrument). The authors performed an XYΩ scan to obtain a hyperspectral image stack, where Ω corresponded to the pump‐Stokes delay (linearly related to the Raman frequency). Each frame was 512 × 512 pixels (dwelling time: 2 µs), and each spectrum sequence had 80 spectral points for SRS imaging within a spectral range of ≈2750–3000 cm^−1^.

Using picosecond pulses for excitation of TPEF, SHG, and TP‐FLIM would result in low contrast and SNR in the images;^[^ ^11^ ^]^ therefore, the authors routed a femtosecond pulse of the pump beam before chirping to expand the nonlinear imaging modalities. The backscattered emission signals of TPEF and SHG were collected by the objective, focused through the lens, separated by the combinations of longpass and bandpass filters (Table S1, Supporting Information), and detected by two PMTs (PMT2101/M, Thorlabs).

FLIM measurements were carried out by synchronously connecting the femtosecond laser, the galvano scanner, and the high‐speed time‐resolved detectors (HPM‐100‐40, Becker & Hickl GmbH) to the TCSPC modules (SPC‐150 and DCC‐100, Becker & Hickl GmbH). The excitation wavelength of the femtosecond beam for TP‐FLIM was the same as that of the pump beam. The lifetime DAQ (DCC‐100, Becker & Hickl GmbH) in TCSPC controlled the gain adjustment of detectors. The authors generated three digital signals (frame pulse, line pulse, and pixel pulse) synchronized with the driver signal of the scanner by a DAQ (PXIe 6341, National Instruments) that were input to the SPC module (SPC‐150 Becker & Hickl GmbH) for photon counting and image reconstruction.

### Control Networks

The hardware control system of the SMILE platform (Figure S1, Supporting Information) was critical for simultaneous multimodal imaging, and an industrial controller based on two DAQs (PXIe‐6115, PXIe‐6341) was core for coordination of logical relationships among the instruments in each imaging system module. In the process of multidimensional information acquisition, the primary function of the controller was to drive XY galvanometers and capture multichannel (TPEF, SHG, SRS) signals simultaneously by two‐channel analog voltage output (PXIe‐6115) and three‐channel analog voltage acquisition (PXIe‐6115). For the SRS module, the function generator outputted a modulated sinusoidal signal, which was amplified by a voltage amplifier to drive the EOM. The SRS signal was demodulated by the lock‐in amplifier with the applied modulated frequency. The authors also used the DAQs to control the motorized linear stage for spectral scanning. In spectral scanning imaging of SRS, a DAQ (PXIe‐6341) generated three synchronous pulse signals (frame pulse, line pulse, and pixel pulse) by three counters that were input to the SPC‐150 and DCC‐100 DAQs. The authors harmonized the logical relationship among the measurement instruments in each module to realize synchronous multi‐nonlinear and fluorescence lifetime imaging for multidimensional information acquisition.

### Calibrations

The spectral‐focusing scheme^[^ ^55^ ^]^ was used to achieve hyperspectral SRS imaging by changing the time‐delay scanning Raman frequency shift. Several standard samples, including DMSO, methanol, and acetic acid, were selected to calibrate the system. The SRS spectra of these standard samples were acquired (Figure S3b, Supporting Information) using spectral scanning by the translation stage (*λ*‐delay) and the spontaneous Raman spectra (Figure S3a, Supporting Information) using a confocal Raman system (Alpha300 R, WITec). Then, the *λ*‐delay position values were converted into Raman frequency shifts based on the central coordinates of some characteristic Raman peaks (Table S2, Supporting Information). The calibration of the spectral‐focusing SRS system was completed by linearly fitting the relationship between the *λ*‐delay and the Raman frequency shift (Figure S3c, Supporting Information).

The authors also benchmark the TP‐FLIM module by repeatedly measuring the lifetime of rhodamine B (Figure S3d, Supporting Information) and rhodamine 6G (Figure S3e, Supporting Information). The synchronization delay between the laser and the acquisition was adjusted until a correct lifetime value was obtained that matched with that measured using a calibrated FLIM system that incorporated a confocal microscope (TCS SP2, Leica) and a TCSPC module. The results showed that the measured fluorescence lifetimes of rhodamine 6G and rhodamine B were ≈4000 and 1600 ps, respectively, approaching their typical values. The same calibrations were applied in the phasor plots (insets in Figure S3d,e, Supporting Information).

### Performance Evaluation

The optical resolution was estimated using 200‐nm‐diameter FBs (f8888, Thermo Fisher). In the process of TPEF imaging using 800 nm excitation, the average resolution determined as the FWHM of the Gaussian function fitting the experimental data was ≈300 nm (*n* = 15), approaching the highest optical resolution at 550 nm emission for a 1.20 NA objective. This resolution was sufficiently high to resolve the characteristics of structural collagen in different types of ovarian carcinoma. Additionally, the spectral resolution (Figure 1c) was measured as the FWHM of the narrowest Raman peak in the DMSO spectrum, which was determined by the linear chirp parameter of two femtosecond pulses and was sufficient to separate different Raman peaks, such as those of lipids and proteins, in CH regions. Additionally, the temporal resolution (Figure 1d) was obtained by measuring the lifetime of the SHG signal of a urea crystal specimen, which approached the IRF of the system. The result demonstrates that the authors' FLIM module was capable of short lifetime measurement and tiny lifetime variation differentiation.

### Statistical Analysis

Since SHG signals were obtained simultaneously with SRS and FLIM data, the SNR could be greatly enhanced by binning N frames into one. The background noise of the SHG images could be reduced by approximately $\sqrt{N}$ (*N* = 80 sequential images for the SRS and FLIM acquisitions).

The spectral stacks of SRS were improved using the authors' previous GSD method.^[^ ^41^ ^]^ Briefly, the pixelwise Raman spectrum in the image stack, *I*_SRS_(*ω*), was modified by convolution with a Lorentzian impulse response, *L*(*ω*), because Raman peaks had Lorentzian envelopes. *L*(*ω*) was given by

(1) $$L\left(\omega \right)=1/\pi \gamma \left[1+{\left(\frac{\omega -\omega_{0}}{\gamma }\right)}^{2}\right]$$

where *ω*_0_ was the central frequency and 2*γ* was the FWHM of *L*(*ω*), which could be set as Ω/3, where Ω could the FWHM of the Raman peak. This linkage between the whole spectral image stack could greatly reduce the noise without causing distortion of the spectrum.

The epithelium and stroma of the ovary were distinguished using the bicomponent cluster method.^[^ ^11^ ^]^ Briefly, the pixelwise decay data of the FLIM images were transformed to Fourier space using the following relations:

(2) $$s_{i,j}\left(\omega \right)=\frac{\int_{0}^{\infty }I(t)\sin(n\omega t)dt}{\int_{0}^{\infty }I(t)dt}$$

(3) $$g_{i,j}\left(\omega \right)=\frac{\int_{0}^{\infty }I(t)\cos(n\omega t)dt}{\int_{0}^{\infty }I(t)dt}$$

where *i* and *j* represented the pixel of the image, sine (s) and cosine (g) were phasor coordinates, *ω*  =  2*πf* (*f* was the laser repetition rate) and *n* was the harmonic frequency. The same components with similar decay signatures formed clusters in the phasor plot.^[^ ^11^ ^]^

All data analyses used raw images, whereas to better show the discernible morphological features for exhibition, the images were regulated by tuning the dynamic range (brightness/contrast) in ImageJ. The fiber angle in image analysis was determined by using the angle tool from the ImageJ toolbar; one side was parallel to the epithelium, and the other side was along the microscopic direction in the angle measurement. For AR analysis, the authors select ROIs mainly in the collagen network in the vicinity of the epithelium in the SHG images for FFT and binarization in ImageJ. The resulting FFT images were subjected to ellipse fitting to calculate the ratio of the major axis using MATLAB. The spectrum extraction from SRS images and the MCR algorithm for multicomponent analysis of spectra were carried out in MATLAB. The lifetime data, including the photon distribution and decay curve over every pixel, were analyzed using SPCImage (Becker & Hickl GmbH). The histograms of free and bound FAD were obtained by separating different tissue constituents via the phasor approach (select cluster). All statistical analyses of the mean, SD, and significant difference (as specified in figure legends) were performed using GraphPad Prism.

## Conflict of Interest

The authors declare no conflict of interest.

## Author Contributions

Y.L. and B.S. contributed equally to this work. Y.L. and B.S. built the system, performed the experiments, and analyzed the data. Y.L. built the control networks. B.S. wrote the data processing algorithms. G.Z. assisted with the multimodal imaging experiments. R.H., L.L., and J.Q. supervised the data analysis. Y.P. provided the tissue samples and performed the pathological classifications. L.L. and J.Q. supervised the project, obtained funding, and edited the manuscript. All authors contributed to writing the manuscript.

## Supporting information

Supporting Information

## Data Availability

The data that support the findings of this study are available from the corresponding author upon reasonable request.
