# Supplementary material for: Super‐Multiplex Nonlinear Optical Imaging Unscrambles the Statistical Complexity of Cancer Subtypes and Tumor Microenvironment
Source: Adv Sci (Weinh). 2021 Dec 19;9(5):2104379. doi: 10.1002/advs.202104379 (PMC8844469; doi:10.1002/advs.202104379)
Supplement: Supplementary file 1 — Supporting Information [file ADVS-9-2104379-s001.pdf]

ADVANCED SCIENCE
Open Access

Supporting Information

for *Adv. Sci.,* DOI: 10.1002/advs.202104379

# Super-multiplex nonlinear optical imaging unscrambles the statistical complexity of cancer subtypes and tumor microenvironment

*Yanping Li, Binglin Shen, Gengjin Zou, Rui Hu, Ying Pan, Junle Qu, Liwei Liu**

# Supplementary Information

## Super-multiplex nonlinear optical imaging unscrambles the statistical complexity of cancer subtypes and tumor microenvironment

Yanping Li[1†], Binglin Shen[1†], Gengjin Zou[1], Rui Hu[1], Ying Pan[2], Junle Qu[1], Liwei Liu[1*]

[1]*Key Laboratory of Optoelectronic Devices and Systems of Guangdong Province and Ministry of Education, College of Physics and Optoelectronic Engineering, Shenzhen University, Shenzhen 518060, China.*

[2]*China–Japan Union Hospital of Jilin University, Changchun, 130033, China.*

[†]These authors contributed equally.

**Supplementary Note 1 Pathological classification and H&E histopathology of ovarian cancer**

Epithelial ovarian cancers (EOCs) are the most common ovarian cancers, accounting for over 90% of ovarian cancers. EOCs are divided into four major types based on histopathology and molecular genetic alterations, including serous (75%), endometrioid (10%), clear-cell (10%), and mucinous (3%) carcinomas[1]. Furthermore, all types of EOC can be subdivided into benign, borderline (intermediate) and malignant (carcinoma) depending on the degree of cell proliferation, nuclear atypia, and the presence of stromal invasion. Because the progress and development of different ovarian cancer types originate from the accumulation of molecular genetic or genomic changes (such as point mutation, gene amplification, deletion and translocation)[1], accurate histological diagnosis is the key to effective treatment of ovarian cancer. Serum cancer antigen (CA-125) levels and histopathological and imaging examinations are widely used in clinical diagnosis. Although an elevated serum CA-125 level is a common symptom in women with stage II and IV ovarian cancer, this level is also affected by other diseases[2] and usually does not increase in borderline or stage I ovarian cancer[3]. Pathological examination, especially H&E histopathology, is a well-accepted gold standard process to diagnose different types of tumors. The H&E histopathology of normal ovarian tissue and four major histological types of ovarian carcinoma are shown in Supplementary Fig. 5, which reveals different pathological morphologies. However, H&E histopathology examination features restricted preparation, which is notoriously time-consuming, labor-intensive, and inherently destructive[4].

**Supplementary Note 2 Ovarian cancer characterization by TPEF and SHG**

In the process of multimodal nonlinear imaging, the abundant collagen fibers in the ECM can produce SHG and TPEF signals, while the epithelial tissue only produces strong TPEF. Therefore, the merged SHG and TPEF image can intuitively distinguish epithelial tissue and ECM according to the tissue morphology (Supplementary Fig. 7). In morphological analysis, the epithelium of normal ovarian tissue features a small thickness, while the epithelium of SBT tissue is thicker. The epithelium of cancer tissue is thickened when the tumor infiltrates into the ECM[5-7].

TACS-1: increased collagen concentration around the tumor and the signature for the presence of locally dense collagen; TACS-2: collagen fibers primarily distribute tangentially along the tumor boundary (approximately 0 deg.); TACS-3: collagen fibers perpendicular to the tumor boundary, with the majority distributed at 90 deg. The collagen fiber angles in normal and SBT tissues (Fig. 3d) are mostly less than 20 deg. (85% and 92%, respectively) corresponding to the TACS-2 type, which is related to the noninvasive characteristics. Conversely, the angle distribution of collagen fibers (TACS-3) in HGSC tissue (Fig. 3m) can reach approximately 90 deg., and the collagen fiber angles in EC and MC tissues (Fig. 3j and Fig. 3p, respectively) are mainly scattered at 40–50 deg.

Binary FFT images of the ROI in the yellow border for the five cancer types are obtained to determine the anisotropy of collagen fibers in the stroma using ellipse fitting. The binary FFT images of normal ovarian and SBT tissues exhibit a more elliptical profile with AR values of 0.37 and 0.49 (Fig. 3e and Fig. 3h), while those of EC, HGSC, and MC tissues are close to a circular contour with AR values of 0.71, 0.90, and 0.87 (Fig. 3k, Fig. 3n, and Fig. 3q, respectively). Fig. 3s shows the statistical analysis results of all the examined samples. The averaged ARs are $0.536 \pm 0.034$, $0.591 \pm 0.035$, $0.717 \pm 0.024$, $0.897 \pm 0.022$, and $0.681 \pm 0.029$ for normal ($n = 10$), SBT ($n = 9$), EC ($n = 9$), HGSC ($n = 10$), and MC ($n = 9$) tissues, respectively. The AR value of SBT shows an extremely significant difference with HGSC ($p < 0.0001$), a significant difference with EC ($p = 0.0093$), and no difference with MC ($p = 0.0671$). For different ovarian carcinomas, the AR values of EC and MC differ from that of HGSC ($p < 0.0001$), whereas there is no discrepancy between EC and MC ($p = 0.36$).

**Supplementary Note 3 Ovarian cancer characterization by SRS**

Altered metabolic activities of lipids, proteins, and other biomolecules in cells and tissue are principal considerations for most cancer studies. The SRS imaging technique is practical for the clinical diagnosis of different types of cancers, such as intracranial, skin, breast, rectal, bladder, prostate and lung tumors[8-12], with great sensitivity resolution, imaging speed, and contrast[13, 14]. We comparatively analyze the content intensity of the five types of ovarian cancer on average across ten tissues for each type. As shown in Fig. 4d, the normal and SBT tissues have higher content intensities than the malignant tumor tissues (EC, HGSC, and MC). Among them, MC tissue exhibits the highest content intensity due to the mucin component found in cytoplasmic mucinous cells[15]. Thus, the difference of the content intensity can be an effective method to obtain cancer diversity.

**Supplementary Note 4 Ovarian cancer characterization by TP-FLIM**

The mean fluorescence lifetimes ($\tau_m = a_1\tau_1 + a_2\tau_2$) of the epithelium (Fig. 5c) and stroma (Fig. 5e) in the normal ovarian specimen (EP: 1.28 ± 0.008 ns, n = 10; ST: 1.7 ± 0.008 ns, n = 10) are significantly higher than those in SBT (EP: 1.06 ±0.018 ns, n = 9; ST: 1.18 ±0.017 ns, n = 9), EC (EP: 0.86 ±0.012 ns, n = 9; ST: 0.99 ±0.013 ns, n = 9), and HGSC (EP: 1.1 ±0.01 ns, n = 12; ST: 1.2 ±0.012 ns, n = 14) specimens, associated with cells of differing metastatic potential. Nevertheless, in all samples evaluated, we observe higher $\tau_m$ values in the epithelium (1.5 ±0.021 ns, n = 9) and stroma (1.7 ±0.03 ns, n = 7) of MC tissue due to the mucin component found in cytoplasmic mucinous cells[15]. There is an extremely significant difference in lifetime ($p < 0.001$) between different ovarian cancer tissues except for HGSC and SBT.

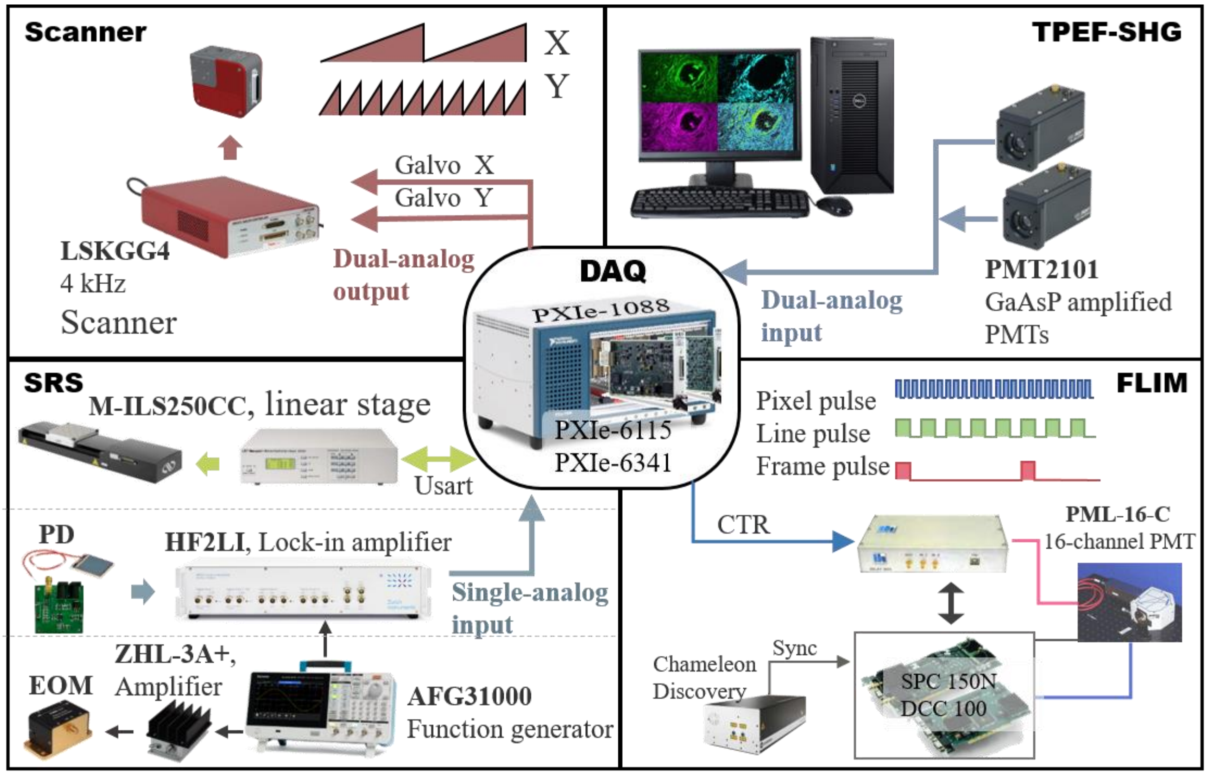


**Supplementary Figure 1 Diagram of controlling system in SMILE platform.** The Industrial controller equipped with two DAQS (PXIe-6115, PXIe-6341) output dual-analog to drive XY galvanometers (LSKGG4, Thorlabs) and acquire multi-channel (TPEF, SHG, SRS) signal concurrently. The signal of TPEF and SHG are detected by two GaAsP amplified PMTs (PMT2101, Thorlabs) and the SRS signal is detected by PD and demodulated with Lock-in amplifier (HF2LI, Zurich Instruments). In SRS module, the controller driver the linear electric translation table (M-ILS250CC, National Instruments) for time delay and spectral scanning. In FLIM module, the FLIM signal is collected by high-speed time-resolved detectors (PML-16-C, Becker & Hickl GmbH), and the controller generates frame pulse, line pulse, pixel pulse signals by three counters to SPC&DCC (SPC-150 and DCC-100, Becker & Hickl GmbH) for the reconstruction of FLIM image.

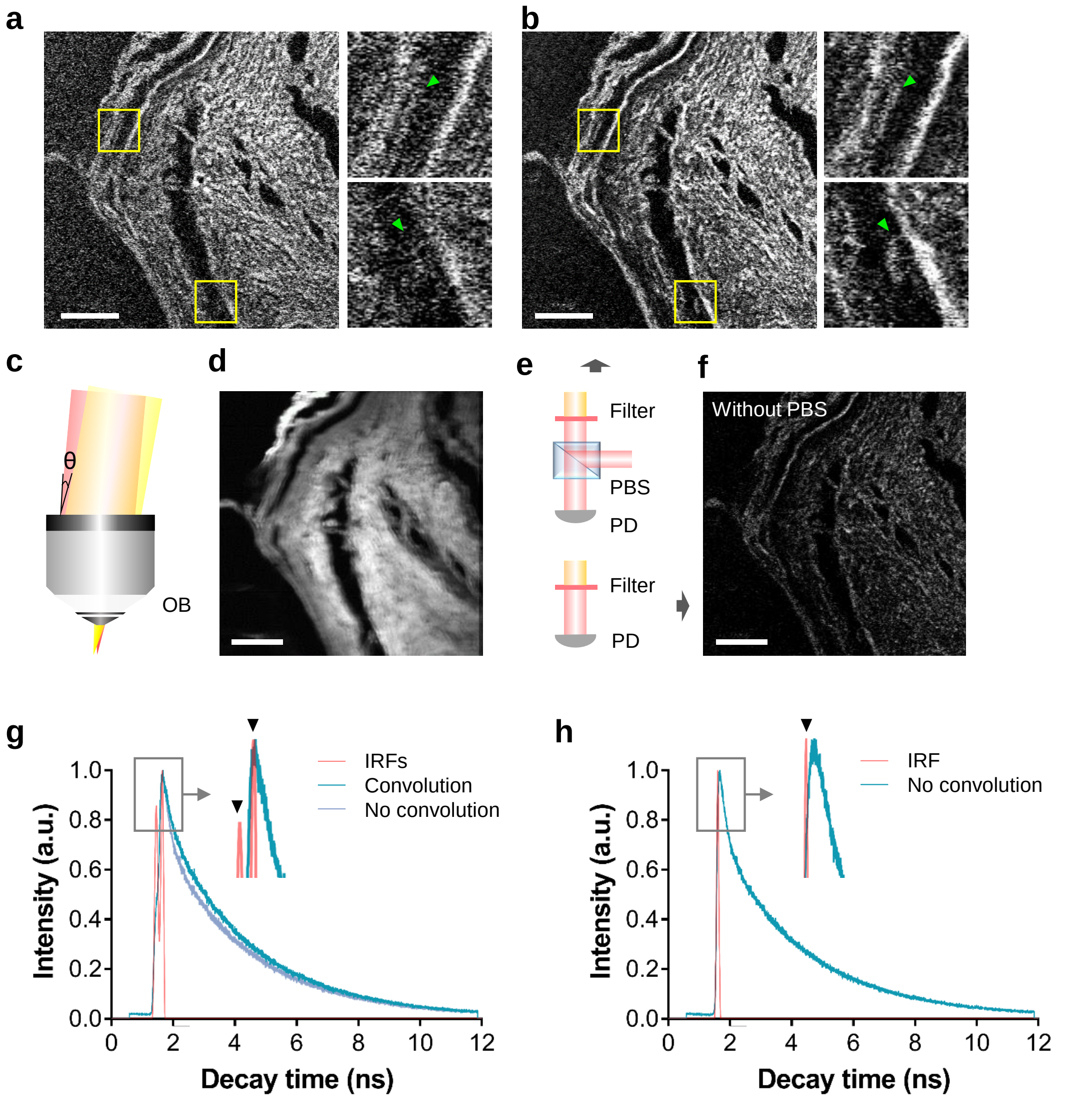


**Supplementary Figure 2 Excitation interference between different imaging modalities.** (a) SRS image with low signal-to-noise ratio (SNR) and contrast caused by the incomplete spatial overlap of the pump picosecond beam and the Stokes picosecond beam. (b) SRS image with high SNR and contrast due to the complete overlap of different light beams. The yellow sqaures indicate the ROIs magnified on the right. The green triangles indicate the resovled details. (c) A small angle (θ) between the femtosecond beam and the picosecond beams can result in image ghosting. (e) Up: using a PBS to block the detection of the vertically polarized femtosecond pulse results in the high SRS intensity in (b). Bottom: not blocking femtosecond pulse before it enters the PD results in the low SRS intensity in (f). (g) Convoluted fluorescence lifetime decay curve in the condition of large time interval between the femtosecond pulse and the picosecond pulses with dual IRFs. (h) Fluorescence lifetime decay curve when the time interval between femtosecond pulse and the picosecond pulses is selected larger than the pulsewidth of the picosecond pulses and smaller than the temporal resolution of the TCSPC.

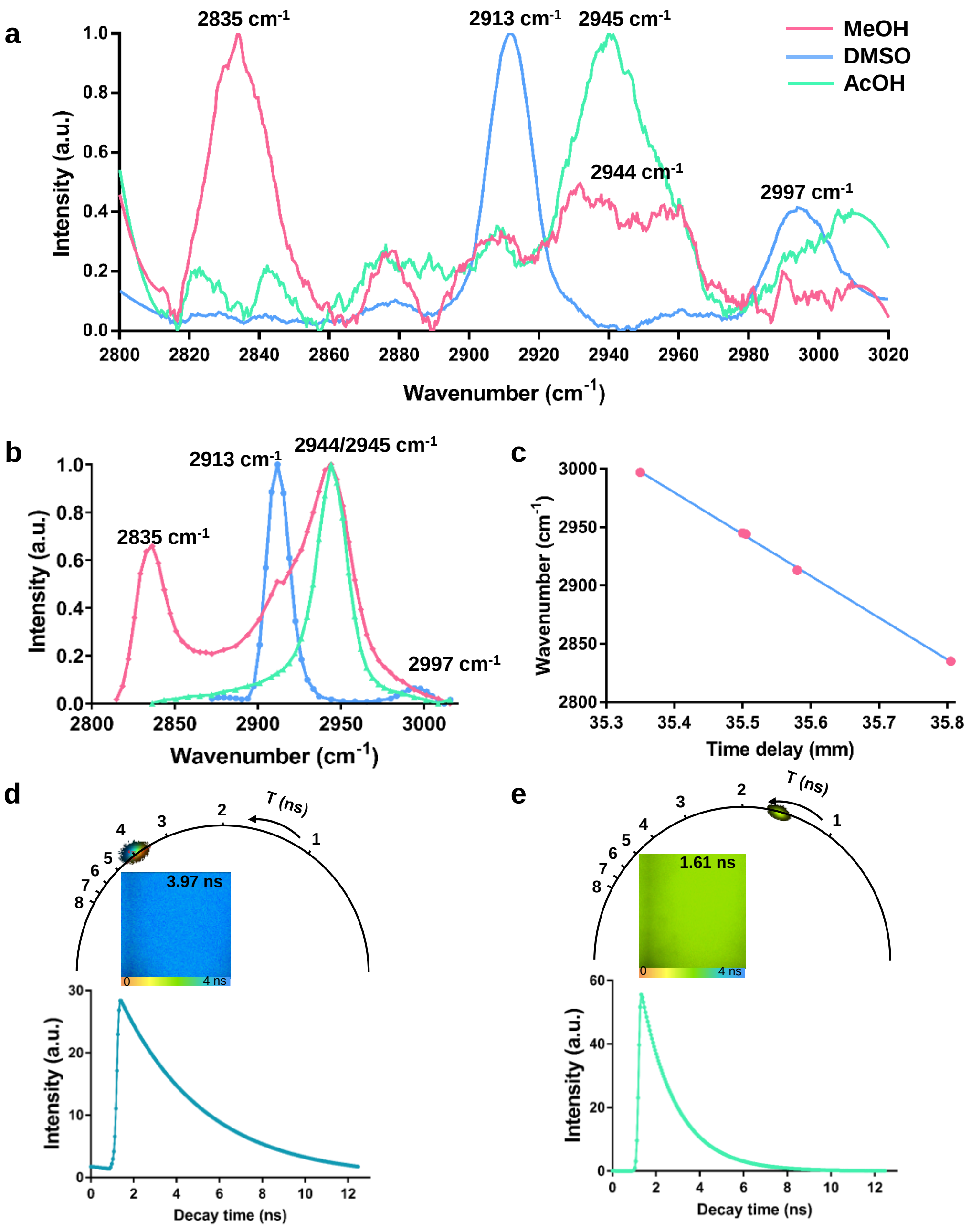


**Supplementary Figure 3 Calibration of the system.** (a) Spontaneous Raman spectra of different solutions. (b) SRS spectra of the same solutions in (a). (c) Correlation between the Raman shift and the pump-Stokes delay. Fluorescence lifetime decays and images and the corresponding phase plots of aqueous solutions of Rhodamine B (d) and Rhodamine 6G (e).

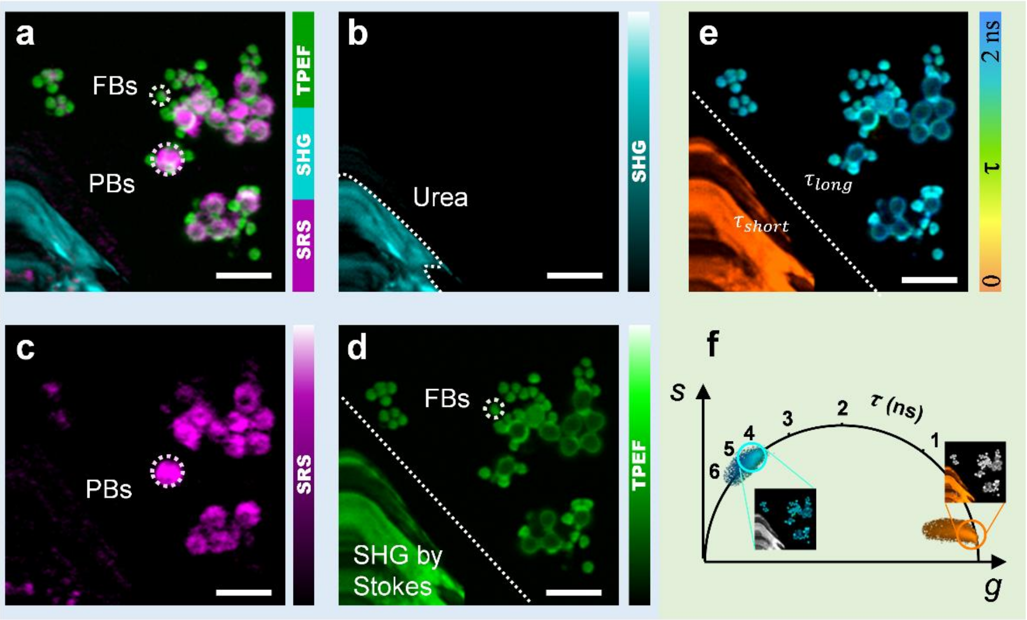


**Supplementary Figure 4 Multimodal contrast imaging of multicomponent mixture.** (a) Simultaneous contrast imaging of FBs, polystyrene beads (PBs), and urea by SHG (b), SRS (c), and TPEF (d), respectively. TP-FLIM (e) with pseudocolor representing the mean lifetime range (indicated by the color bar) and the corresponding phasor plot (f) distinguish different components via the lifetime contrast ($\boldsymbol{\tau_{short}}$ and $\boldsymbol{\tau_{long}}$). The color bar in (a) represents the pseudocolor of the three modalities, the color bar in (b)–(d) represent the normalized intensity. Scale bars, 10 μm.

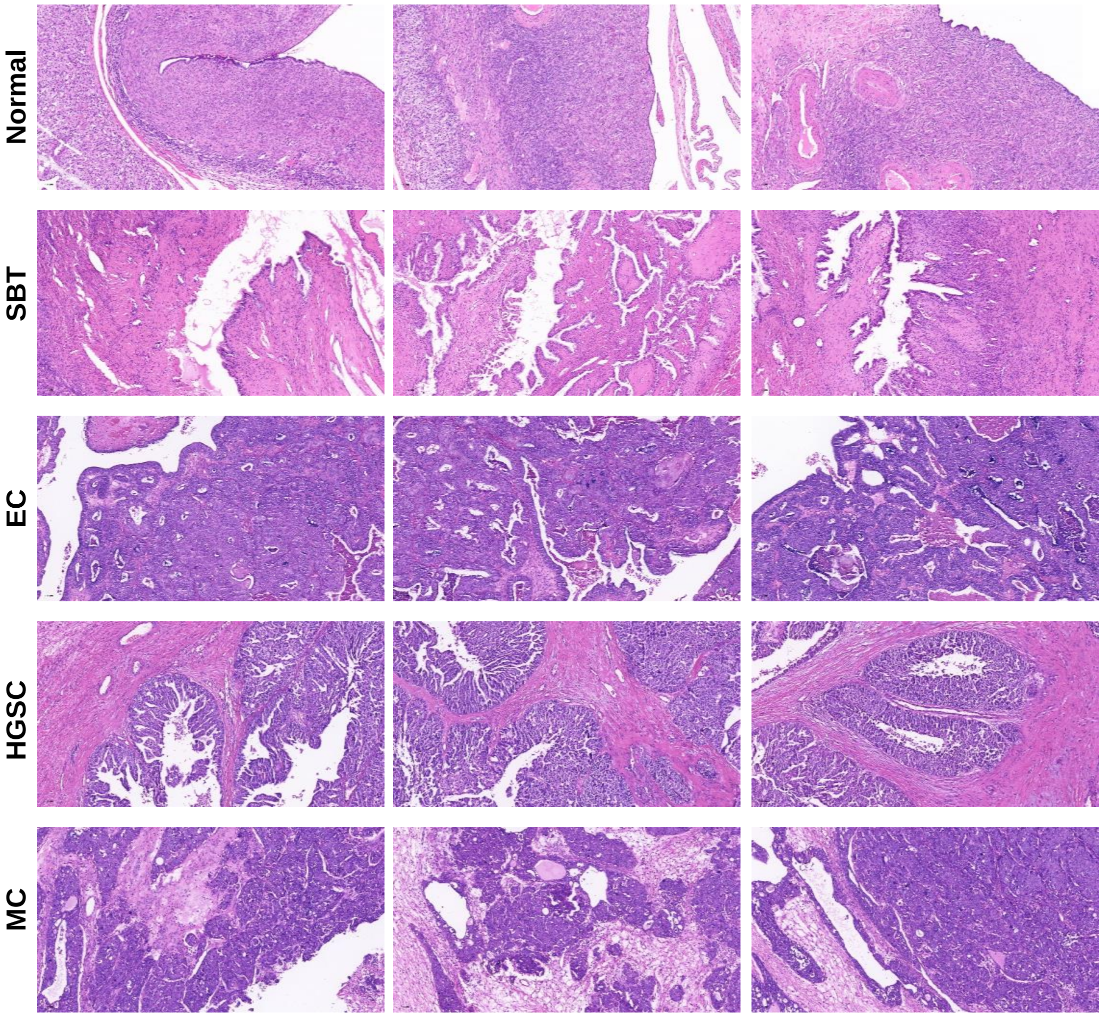


**Supplementary Figure 5 Representative examples of the major histological types of ovarian carcinoma.** SBT: serous borderline tumor; EC: endometrioid carcinomas; HGSC: high-grade serous carcinomas; MC: mucinous carcinomas.

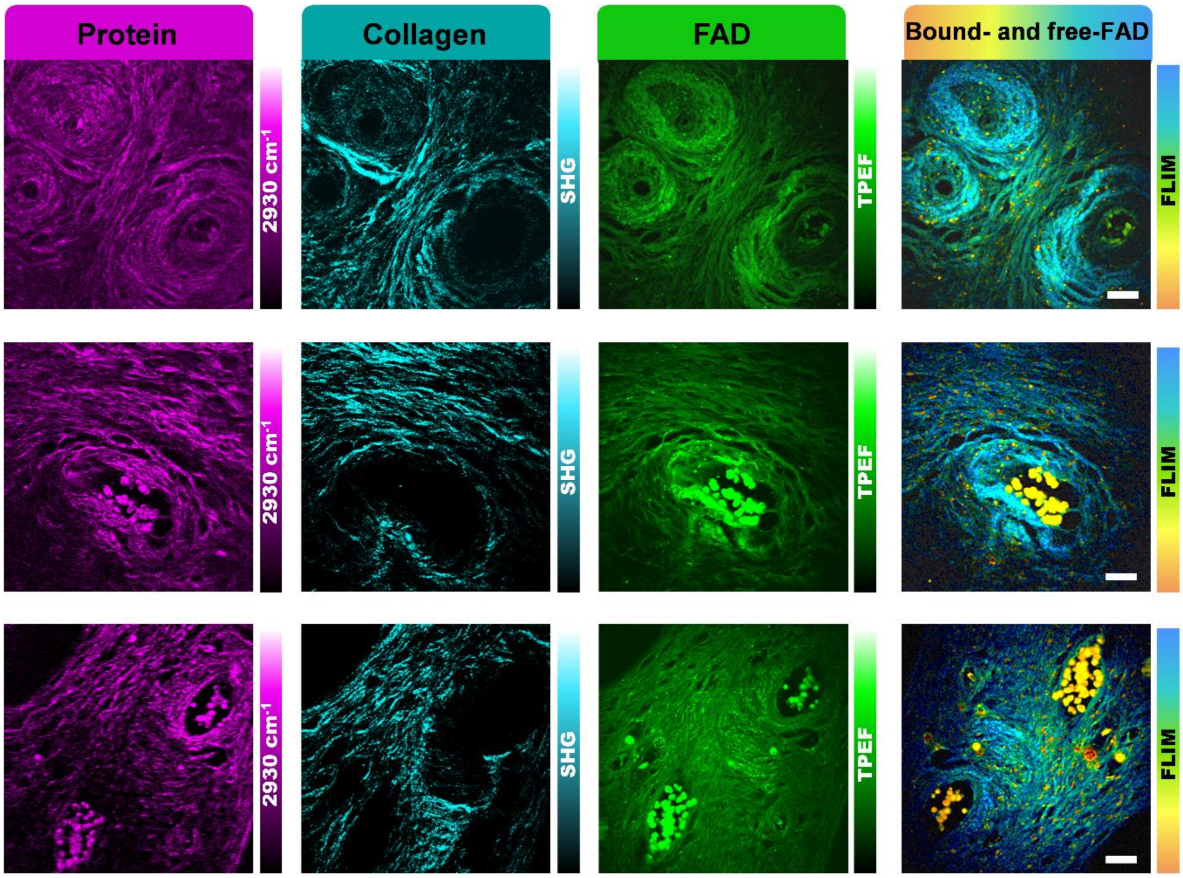


**Supplementary Figure 6 Simultaneous multimodal imaging of human ovarian tissues.** First to fourth column correspond to SRS, SHG, TPEF and FLIM imaging. Up to down correspond to different pathological tissues of EC, HGSC, and MC. The color bar in the SRS, SHG, and TPEF images represent the normalized intensity, and those in the FLIM images represent the lifetime range. Scale bar, 20 μm.

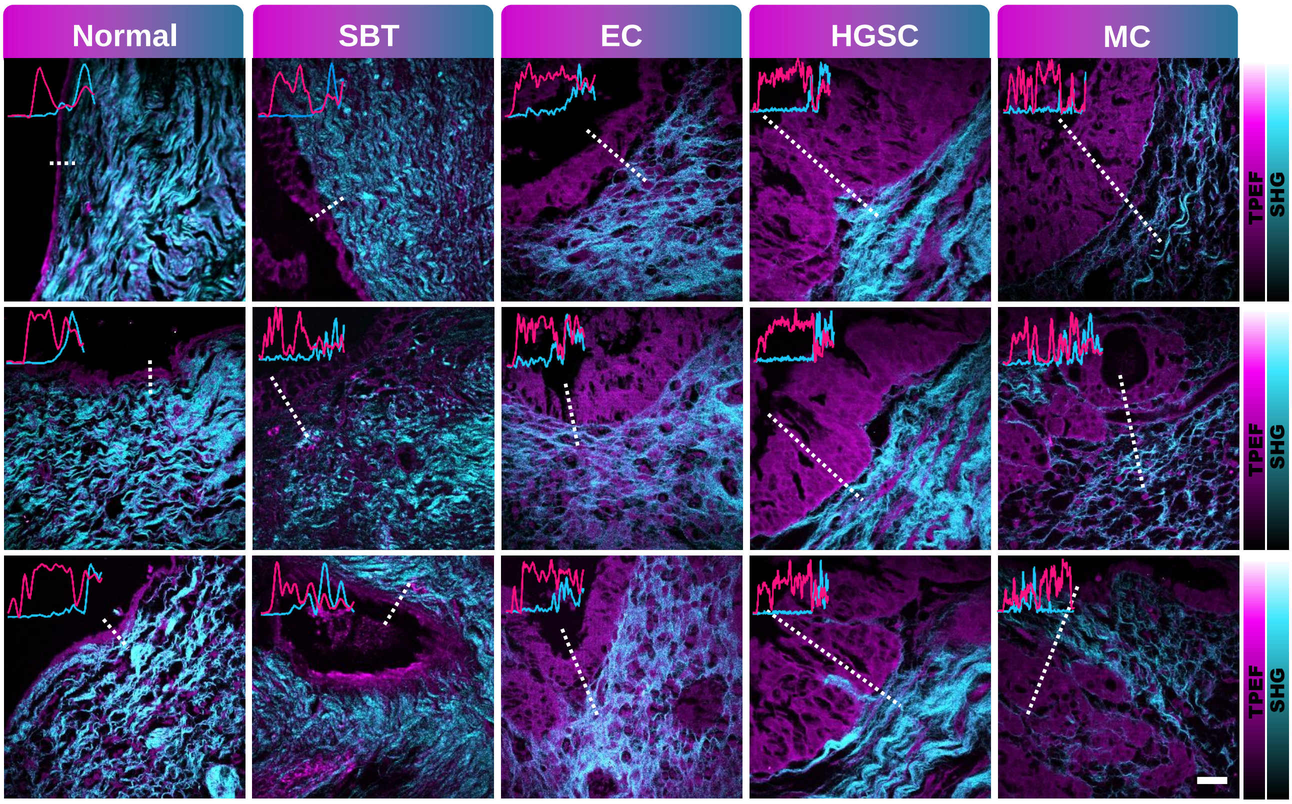


**Supplementary Figure 7 SHG/TPEF image analysis of different histological types of ovarian carcinoma.** cyan-SHG (collagen fibers); magenta-TPEF (FAD). The magenta and cyan curves reveal the infiltration thickness of epithelial cancer cells in different pathological types of ovarian cancer tissues from tissue morphology. Scale bar, 20 μm.

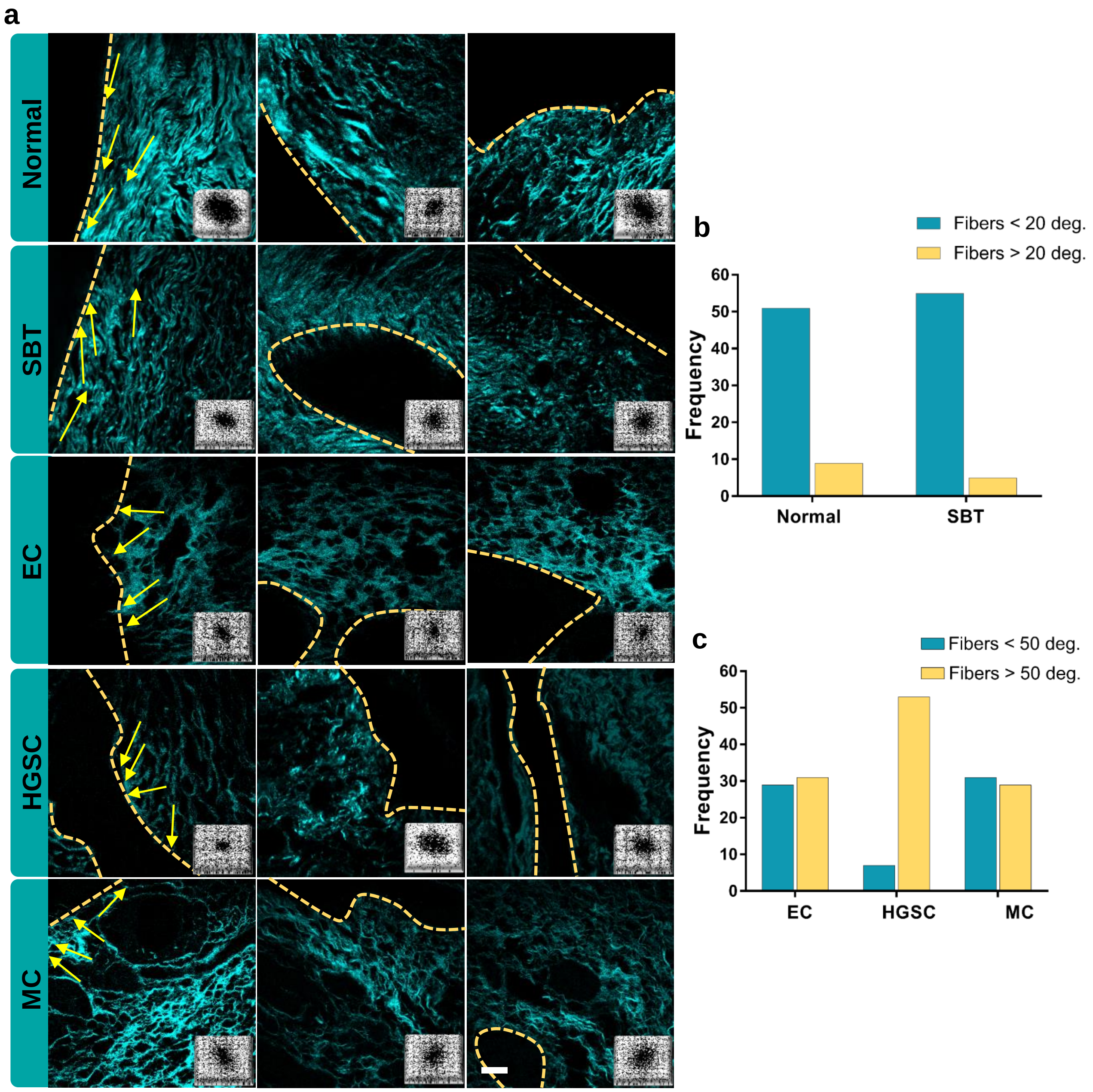


**Supplementary Figure 8 Quantitative analysis of collagen in different pathological types of ovarian tissue from SHG image.** (a) Analysis of collagen fiber (yellow arrow) angles relative to the epithelial boundary (yellow line) and Fourier diagram (illustration at the right bottom) for normal ovary, SBT, EC, HGSC, and MC. (b) Quantitative analysis of fibers collagen fibers that are principally distributed around 0 deg (fibers angle < 20 deg.) in normal and SBT samples. (c) Quantitative analysis of collagen fibers that are disorderly distributed (all directions) in EC and MC samples, and collagen fibers in HGSC are distributed are around 70 deg (fibers angle > 50 deg.). Scale bar, 20 μm.

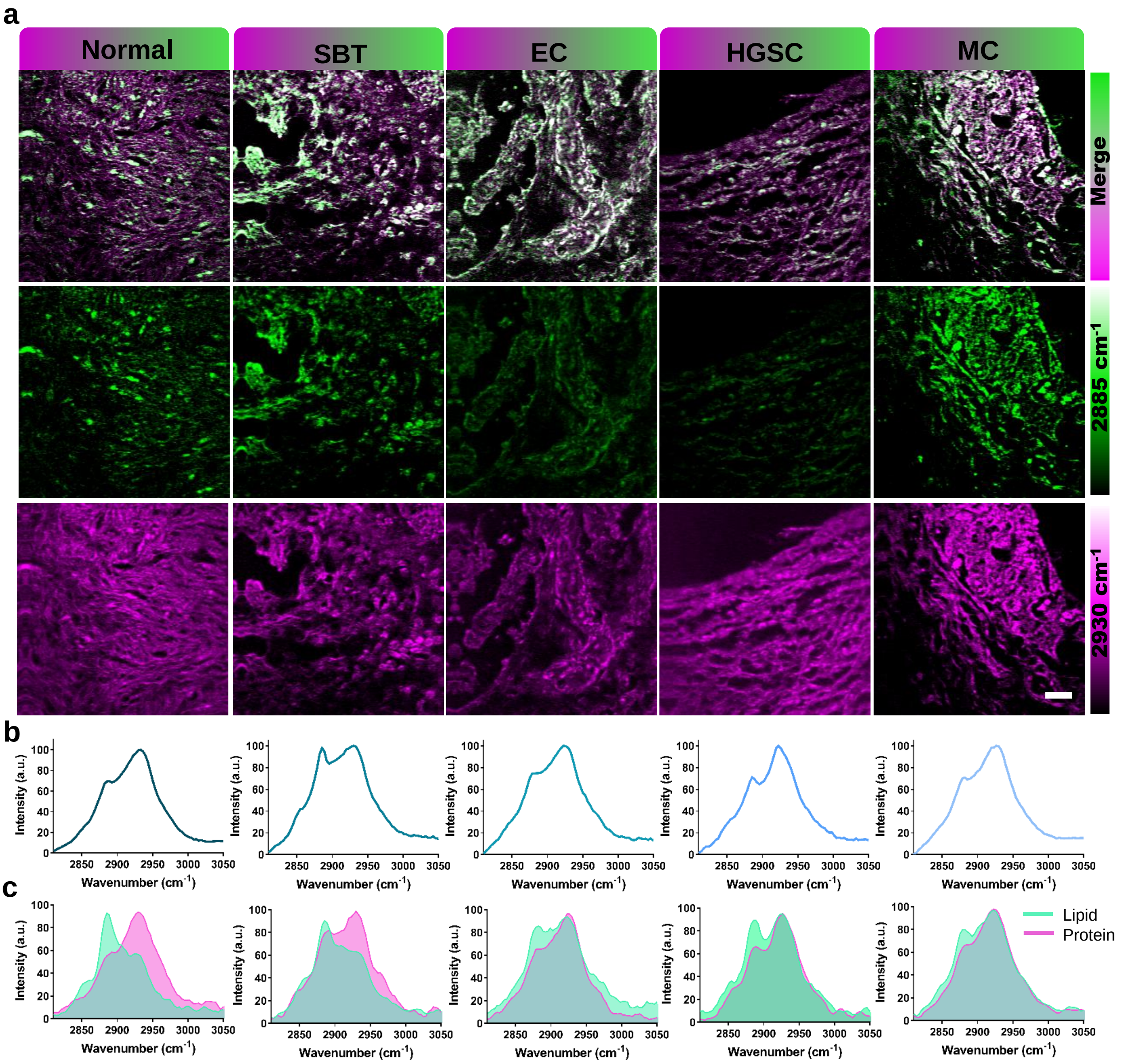


**Supplementary Figure 9 Hyperspectral SRS imaging of lipid and protein in cancerous ovarian tissue.** (a) Merged SRS images (Top) reveal (Top) the distribution of lipid (green, middle) and protein (magenta, bottom) in different pathological types of ovarian tissue. (b) Overall SRS spectra and (c) spectra retrieved of lipid (green), and protein (magenta) in normal and cancerous liver tissues. Scale bar, 20 μm.

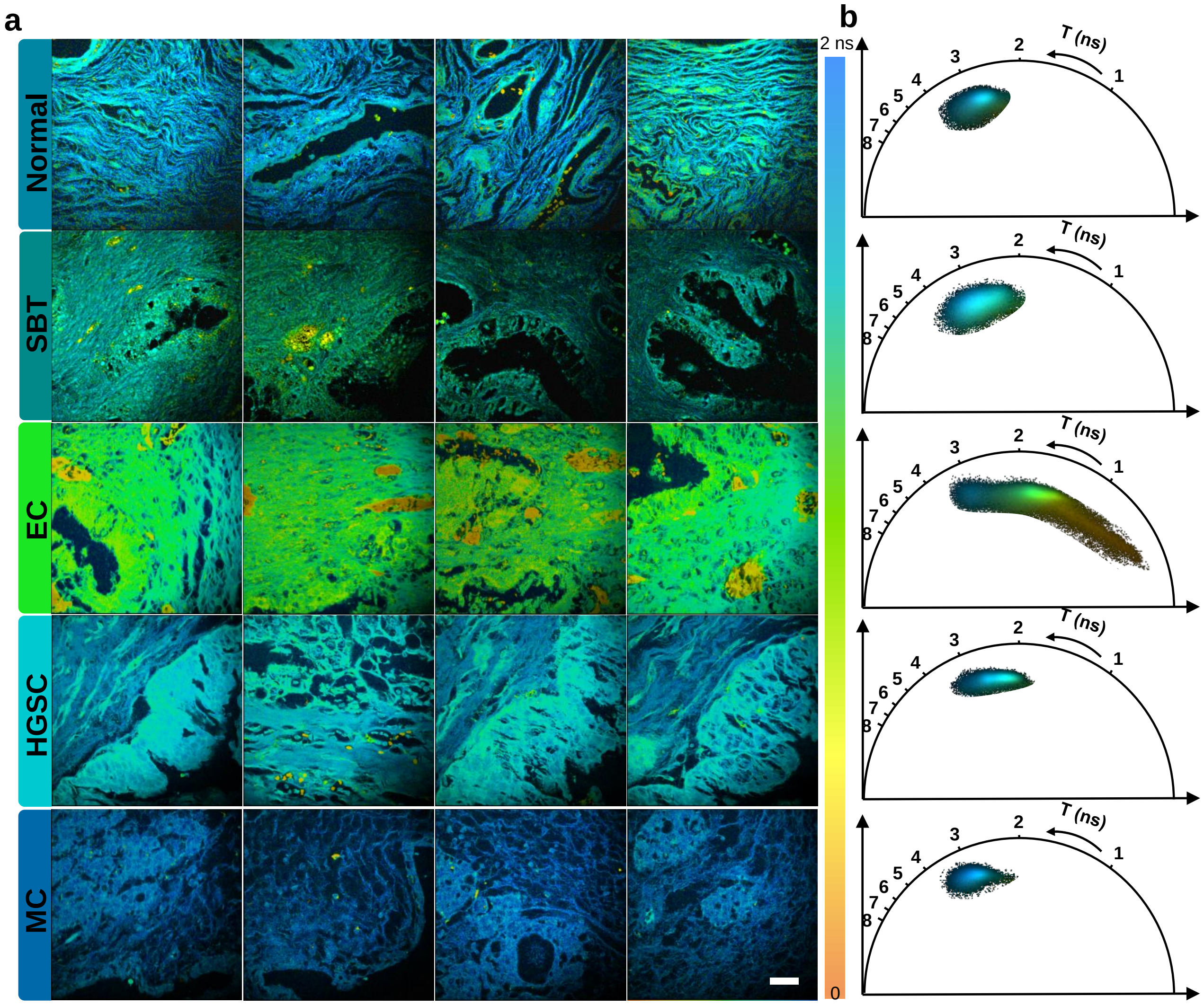


**Supplementary Figure 10 FLIM and phasor analysis of energy metabolism in ovarian tissue.** (a) Fluorescence lifetime (pseudocolor) images and (b) Phasor analysis of endogenous fluorescence (FAD) in different pathological types of ovarian tissue. Scale bar, 20 μm.

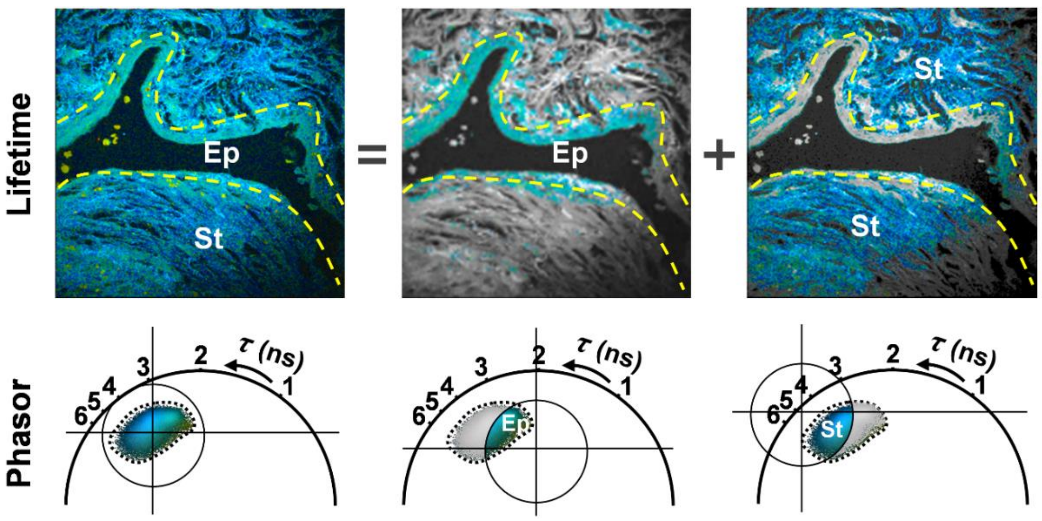


**Supplementary Figure 11 Phasor approach to distinguish different tissue constituents using cluster method.** The cursors in the phasor plots (bottom row) correspond to the color-mapped constituents of Ep and St in the lifetime images (upper row).

**Supplementary Table 1 Specified dichroic mirrors and bandpass filters in SMILE platform.**

| Laser (nm) | LP (nm) | BP (nm) | Modality | Object |
|---|---|---|---|---|
| 800 (fs) | 750 | 550/80 | TPEF | FAD |
| | | 550/40 | TPEF-FLIM | FAD |
| | 500 | 400/10 | SHG | Collage |
| 800 (ps)<br>1040 (ps) | / | 825/150 | SRS | Lipid<br>Protein |

**Supplementary Table 2 Raman peaks used for the calibration of the spectral focusing system.**

| Specimens | Raman Peak ($cm^{-1}$) | Time delay (mm) |
|---|---|---|
| DMSO | 2913 | 35.58 |
| | 2997 | 35.35 |
| MeOH | 2945 | 35.5 |
| | 2835 | 35.805 |
| AcOH | 2944 | 35.505 |

**Supplementary Table 3 Statistics of component lifetime and ratio of free and bound FAD**

| | EP | | | | ST | | | |
|---|---|---|---|---|---|---|---|---|
| | n | $\tau_{bound}$ (ps) | $\tau_{free}$ (ps) | $R_{bound}$ | n | $\tau_{bound}$ (ps) | $\tau_{free}$ (ps) | $R_{bound}$ |
| Normal | 10 | 317 ±6 | 1980 ±12 | 42 ±1 | 10 | 334 ±6 | 2161 ±13 | 37 ±1 |
| SBT | 9 | 334 ±9 | 1763 ±25 | 48 ±1 | 9 | 345 ±9 | 1920 ±16 | 46 ±1 |
| EC | 9 | 324 ±36 | 1575 ±15 | 56 ±1 | 9 | 339 ±4 | 1726 ±23 | 53 ±1 |
| HGSC | 12 | 316 ±3 | 2045 ±19 | 54 ±1 | 14 | 313 ±5 | 2201 ±18 | 57 ±1 |
| MC | 9 | 365 ±27 | 2121 ±19 | 35 ±1 | 7 | 493 ±55 | 2281 ±20 | 29 ±1 |

**Supplementary References**


[1] K. R. Cho, I. M. J. A. R. o. P. Shih, Annual Review of Pathology **2009**, 4, 287.
[2] S. Milman, K. D. Whitney, N. Fleischer, Thyroid **2011**, 21, 913.
[3] G. Mor, I. Visintin, Y. Lai, H. Zhao, P. Schwartz, T. Rutherford, L. Yue, P. Bray-Ward, D. C. Ward, Proceedings of the National Academy of Sciences of the United States of America **2005**, 102, 7677.
[4] H. H. Tu, Y. Liu, D. Turchinovich, M. Marjanovic, J. K. Lyngso, J. Laegsgaard, E. J. Chaney, Y. B. Zhao, S. X. You, W. L. Wilson, B. W. Xu, M. Dantus, S. A. Boppart, Nature Photonics **2016**, 10, 534.
[5] D. D. Bowtell, S. Bohm, A. A. Ahmed, P. J. Aspuria, R. C. Bast, V. Beral, J. S. Berek, M. J. Birrer, S. Blagden, M. A. Bookman, J. D. Brenton, K. B. Chiappinelli, F. C. Martins, G. Coukos, R. Drapkin, R. Edmondson, C. Fotopoulou, H. Gabra, J. Galon, C. Gourley, V. Heong, D. G. Huntsman, M. Iwanicki, B. Y. Karlan, A. Kaye, E. Lengyel, D. A. Levine, K. H. Lu, I. A. McNeish, U. Menon, S. A. Narod, B. H. Nelson, K. P. Nephew, P. Pharoah, D. J. Powell, P. Ramos, I. L. Romero, C. L. Scott, A. K. Sood, E. A. Stronach, F. R. Balkwill, Nat Rev Cancer **2015**, 15, 668.
[6] S. Lheureux, M. Braunstein, A. M. Oza, Ca-Cancer J Clin **2019**, 69, 280.
[7] J. J. A. o. O. Prat, Annals of Oncology **2012**, 23 Suppl 10, x111.
[8] H. P. Li, Y. Ren, D. L. Song, F. Yu, S. Y. Jiang, S. Wang, Eleventh International Conference on Information Optics and Photonics (Ciop 2019) **2019**, 11209.
[9] L. H. Li, Z. F. Chen, X. F. Wang, X. Liu, W. Z. Jiang, S. M. Zhuo, L. W. Jiang, G. X. Guan, J. X. Chen, Oncotarget **2017**, 8, 107323.
[10] E. Baria, S. Morselli, S. Anand, R. Fantechi, G. Nesi, M. Gacci, M. Carini, S. Serni, R. Cicchi, F. S. Pavone, Journal of Biophotonics **2019**, 12.
[11] R. E. Kast, S. C. Tucker, K. Killian, M. Trexler, K. V. Honn, G. W. Auner, Cancer Metast Rev **2014**, 33, 673.
[12] X. Y. Xu, J. Cheng, M. J. Thrall, Z. F. Liu, X. Wang, S. T. C. Wong, Biomed Opt Express **2013**, 4, 2855.
[13] L. L. Zhang, Y. Z. Wu, B. Zheng, L. Z. Su, Y. Chen, S. Ma, Q. Q. Hu, X. Zou, L. Yao, Y. L. Yang, L. Chen, Y. Mao, Y. Chen, M. B. Ji, Theranostics **2019**, 9, 2541.
[14] S. Yan, S. S. Cui, K. Ke, B. X. Zhao, X. L. Liu, S. H. Yue, P. Wang, Analytical Chemistry **2018**, 90, 6362.
[15] J. Adur, V. B. Pelegati, A. A. de Thomaz, M. O. Baratti, D. B. Almeida, L. A. L. A. Andrade, F. Bottcher-Luiz, H. F. Carvalho, C. L. Cesar, Plos One **2012**, 7.
